# Characterization of a Thermostable Lichenase from *Bacillus subtilis* B110 and Its Effects on β-Glucan Hydrolysis

**DOI:** 10.4014/jmb.2111.11017

**Published:** 2021-12-15

**Authors:** Zhen Huang, Guorong Ni, Fei Wang, Xiaoyan Zhao, Yunda Chen, Lixia Zhang, Mingren Qu

**Affiliations:** 1 Key Laboratory of Animal Nutrition of Jiangxi Province, Nutritional Feed Development Engineering Research Center, Jiangxi Agricultural University, Nanchang, Jiangxi 330045, P.R. China; 2 College of Land Resources and Environment, Jiangxi Agricultural University, Nanchang, Jiangxi 330045, P.R. China; 3 College of Bioscience and Bioengineering, Jiangxi Agricultural University, Nanchang, Jiangxi 330045, P.R. China

**Keywords:** *Bacillus subtilis*, lichenase, expression, characterization, oligosaccharide, β-glucan

## Abstract

Lichenase is an enzyme mainly implicated in the degradation of polysaccharides in the cell walls of grains. Emerging evidence shows that a highly efficient expression of a thermostable recombinant lichenase holds considerable promise for application in the beer-brewing and animal feed industries. Herein, we cloned a lichenase gene (*CelA203*) from *Bacillus subtilis* B110 and expressed it in *E. coli*. This gene contains an ORF of 729 bp, encoding a protein with 242 amino acids and a calculated molecular mass of 27.3 kDa. According to the zymogram results, purified CelA203 existed in two forms, a monomer, and a tetramer, but only the tetramer had potent enzymatic activity. CelA203 remained stable over a broad pH and temperature range and retained 40% activity at 70°C for 1 h. The *K*_m_ and *V*_max_ of CelA203 towards barley β-glucan and lichenan were 3.98 mg/ml, 1017.17 U/mg, and 2.78 mg/ml, 198.24 U/mg, respectively. Furthermore, trisaccharide and tetrasaccharide were the main products obtained from CelA203-mediated hydrolysis of deactivated oat bran. These findings demonstrate a promising role for CelA203 in the production of oligosaccharides in animal feed and brewing industries.

## Introduction

β-1,3-1,4-glucans are linear polysaccharides mainly found in the cell wall of a wide range of cereal plants [1]. They are composed of about 1200 β-D-glucose residues linked by β-1,3 and β-1,4 glycosidic bonds, with the ratio of 1:3 [2]. Previous evidence has revealed mixed-linkage β-glucan in *Cetraria islandica*, and the ratio of β-1,3 and β-1,4 glycosidic bonds was about 1:1 [3]. β-glucanases mainly contribute to the hydrolysis of β-glucans and are classified into four groups according to different types of broken bonds. These include cellulase (endo-1,4-β-D-glucanases, E.C. 3.2.1.4), endo-1,4-β-D-glucanase (E.C. 3.2.1.6), lichenase (β-1,3-1,4-glucanase, E.C. 3.2.1.73) and laminarinase (β-1,3-glucanase, E.C. 3.2.1.39) [4, 5, 6].

Plants and microorganisms both produce lichenases. Microbial lichenases belong to glycoside hydrolase family 16 [7]. Previous investigations have isolated, cloned, and characterized a number of lichenases from *Bacillus* species, including *B. pumilus* [8], *B. subtilis* [9-11], *B. altitudinis* [12], and *B. tequilensis* [13]. In addition, several non-*Bacillus* species have been studied, including *Paenibacillus barengoltzii* [14], *Paenibacillus barcinonensis* BP-23 [15], *Penicillium occitanis* Pol6 [16], *Thermoascus aurantiacus* [17], *Aspergillus awamori* [18], and *Aspergillus niger* US368 [19].

Lichenases are widely used in animal feed and the brewery industries. For instance, in breweries, lichenases are used to increase the leaching rate and reduce colloidal precipitation in beer as it reduces the viscosity of malt [14,16,18]. Moreover, the addition of β-glucanases during the pelletizing process of monogastric animal feedstuff improves feed digestibility and reduces the health problems associated with fecal stickiness [7]. In a previous study, oat β-glucan produced by the action of lichenase was revealed to promote nutrient absorption and exert antihyperlipidemic effects [13]. These findings are intriguing and warrant the development of a novel lichenase with noticeable thermostability and high levels of expression to meet industrial needs. In this study, we cloned and characterized (biochemically) a lichenase gene from the GH family 16 in *B. subtilis* B110. The potential of the lichenase to hydrolyze oat bran into oligosaccharides was also studied.

## Materials and Methods

### Chemicals, Strains, Media and Plasmids

Barley β-glucan (80% purity), laminarin, carboxymethyl cellulose (CMC), xylan, curdlan, avicel, and chitosan were purchased from Shanghai McLean Biochemical Technology Co., Ltd. Lichenin was purchased from Megazyme (Ireland).

*Bacillus subtilis* B110 strain was acquired from the Engineering Laboratory for the Development and Utilization of Agricultural Microbial Resources of Jiangxi Province and maintained in a lysogeny broth. *Escherichia coli* DH5α (Invitrogen Co., China) was used as the host strain for gene manipulation. *E. coli* BL21 (DE3) (Novagen Co., China) was used for recombinant expression. Plasmid pET-29a (TakaRa Biotechnology, China) served as the expression vector.

### Gene Cloning, Sequencing, and Construction of the Expression Vector

Genomic DNA was extracted from *Bacillus subtilis* B110 cells following a previously described protocol [20]. After whole genome sketch sequencing of *B. subtilis* B110, a putative gene encoding the lichenase was cloned by PCR amplification using the following primers: *CelA203*F (5′- TAAGAAGGAGATATACATATGCCTTATCTGAAACGAGTGTT-3′(the underline points to the restriction enzyme cutting site) and CelA203R (5′-GTGGTGGTGGTGGTGCTCGAGTTTTTTTGTATAGCGCAC -3′). The resultant PCR product was purified using a gel extraction kit (BioTeke Co., China). After digestion with two restriction endonucleases (*Nde*I and *Xho*I), the pET29a (+) vector with a 6× His-tag at the C-terminal was ligated using a one-step cloning kit (Vazyme Biotech, China). Thereafter, the positive transformants were sequenced by Tsingke Corporation (China).

The isoelectric point and molecular mass were predicted using an online tool (https://web.expasy.org). The CelA203 protein sequence was determined via the NCBI database (http://blast.ncbi.nlm.nih.gov/). Multiple alignments of sequences were performed with the ClustalW program in the Mega 5.0 software [21]. Subsequently, phylogenetic analysis was performed via the Poisson model adjacency method.

### Expression and Purification of CelA203

The expression host, *E. coli* BL21 (DE3), was cultured in LB media (supplemented with kanamycin, final concentration 50 μg/ml) at 37°C until the OD (600 nm) reached 0.5-0.6. Next, 0.2 mM (final concentration) IPTG was added at 16°C for 24 h. Cells of the recombinant strain were centrifuged at 8,000 g for 20 min, then resuspended in 20 mM Tris-HCl and 100 mM NaCl at a pH of 7.0, sonicated (Xinzhi Co., China), and centrifuged again (12,000 ×*g*, 10 min, 4°C). After that, the crude protein extract was loaded onto a Ni-NTA column and then eluted with Tris-HCl (pH 7.0) containing different concentrations of imidazole (50,100, 150, 200, and 300 mM). The collected samples were analyzed in 10% SDS-PAGE [22]. The protein concentration was determined following a previously described method [23].

### Zymogram Analysis of Purified CelA203

To conduct SDS-PAGE, 0.1% sodium CMC was added directly into the separation gel. After electrophoresis, the gels were soaked in Triton X-100 (2.5%, v/v) twice for 30 min, washed thoroughly with Tris-HCl buffer (pH 8.0), and incubated in 20 mM sodium phosphate buffer (pH 7.0) at 50°C for 10 min. Subsequently, the gels were stained with Congo red solution (0.1%) and decolorized with 1M NaCl.

### Size-Exclusion Chromatography Determination of CelA203

The molecular mass of purified CelA203 was determined by FPLC sieve chromatography (Enrich SEC 650 10 × 300 column, Bio-Rad, USA), and eluted in 20 mM Tris-HCl (pH 7.0) at a rate of 0.5 ml/min. A high-molecular-weight (HMW) Native Electrophoresis Protein Marker Kit (66-669) (Beijing Jiehuibogao Biotechnology Co., Ltd.) was employed to calculate the molecular weight of CelA203.

### Enzyme Assay

According to the dinitrosalicylic acid (DNS) method [24], 1% (w/v) substrate was used to measure lichenase activity. Briefly, 28.68 μg of the pure enzyme was added to sodium phosphate buffer (pH 6.0) containing 0.2%barley β-glucan (w/v) and the mixture was incubated at 50°C for 5 min. An enzyme unit of lichenase was expressed as the amount of enzyme required to produce 1 μmol of reducing sugar (glucose equivalent) per minute.

### Effects of pH and Temperature on the Activity of CelA203

The optimal pH for CelA203 was assessed with 28.68 μg in various 20 mM buffers, including the sodium citrate buffer (pH 3.0-6.0), sodium phosphate buffer (pH 6.0-7.5), Tris-HCl buffer (pH 7.0-9.0) and glycine-NaOH buffer (pH 9-10.5), incubated with 0.2% barley β-glucan at 50°C for 5 min. Furthermore, aliquots of CelA203 were incubated in different buffers (pH 3.0-10.5) for 24 h at 4°C to determine the tolerance to different pH values. After that, the residual enzyme activity was assessed under standard conditions.

CelA203 was incubated for 5 min at different temperatures (4 to 100°C) in sodium phosphate buffer (pH 6.0) to determine the optimal reaction temperature. The thermostability of CelA203 was then measured by depositing the purified enzyme in sodium phosphate buffer at 4-70°C for 48 h.

### Effect of Metal Ions and Compounds on the Activity of CelA203

Exactly 1 mM of various metal irons (Ca^2+^, Zn^2+^, Cu^2+^, Mg^2+^, Fe^2+^, Co^2+^, Ni^2+^, Li^+^, K^+^, Fe^3+^, Cr^3+^, and Mn^2+^) and compounds (Tween-80, DMSO, SDS, EDTA, acetone, ethanol, methanol, acetonitrile, isopropanol, and Triton X-100) were mixed separately with sodium phosphate buffer (pH 6.0). The mixtures were pre-incubated at 50°C for 10 min. Reactants without any additives served as controls whose enzyme activity was considered to be 100%.

### Substrate Specificity of CelA203

To test substrate specificity, 0.5% (w/v) of laminarin, CMC, barley β-glucan, ichenan, curdlan, avicel, xylan, and chitosan were analyzed under optimal condition. The kinetic parameters of the enzyme were obtained by measuring the enzyme activity using various concentrations of barley β-glucan (0.4-2.8 mg/ml) and lichenan (1.2-4.0 mg/ml) at 50°C for 5 min in sodium phosphate buffer (pH 6.0). The *K*_m_ and *V*_max_ values were calculated using the Lineweaver-Burk plot [16].

### Hydrolytic Properties of CelA203

The products of hydrolysis from two substrates treated with CelA203 were verified using TLC. Briefly, 1% (w/v) of substrates and 10 U of the purified enzyme was incubated in 1 ml of optimal buffer at 50°C, and samples were collected at different times. Thereafter, the end product was put in a silicone glass plate (Merck, Germany), and then developed using n-butanol: water: acetic acid (2:1:1 v/v/v) as the developing solvent [14]. Next, samples were air-dried and visualized by spraying in sulfuric/methanol acid (1:4, v/v) followed by heating at 100°C until they were visible. Additionally, glucose, cellobiose, cellotriose, and cellotetraose were measured as standard saccharides.

### CelA203-Mediated Hydrolysis of Oat Bran to β-Gluco-Oligosaccharides

In this experiment, the deactivated endogenous enzyme in oat bran was obtained. Briefly, oat bran was mixed with an aqueous solution of ethanol (80%), distilled, and refluxed at 80°C for 2 h. After that, 1 g of deactivated oat bran or oat bran was blended in 20 ml of 20 mM sodium phosphate buffer buffer (pH 6.0), and then 0 and 104 U of the purified enzyme per gram of oat bran was added, followed by incubation at 50°C for 12 h. The reaction mixtures were withdrawn at various intervals, subsequently boiled for 5 min, and centrifuged at 10,000 g for 10 min. The samples were assessed for reducing sugars by the DNS method [24]. The products of hydrolysis were confirmed using TLC following a similar method to the one described above.

### Gene Registration Number

The lichenase gene (*CelA20*3) sequence, cloned from *B. subtilis* B110, has been submitted to the GenBank database under the accession number MW233898.

## Results

### Analysis of the CelA203 Sequence

**Fig. 1:**
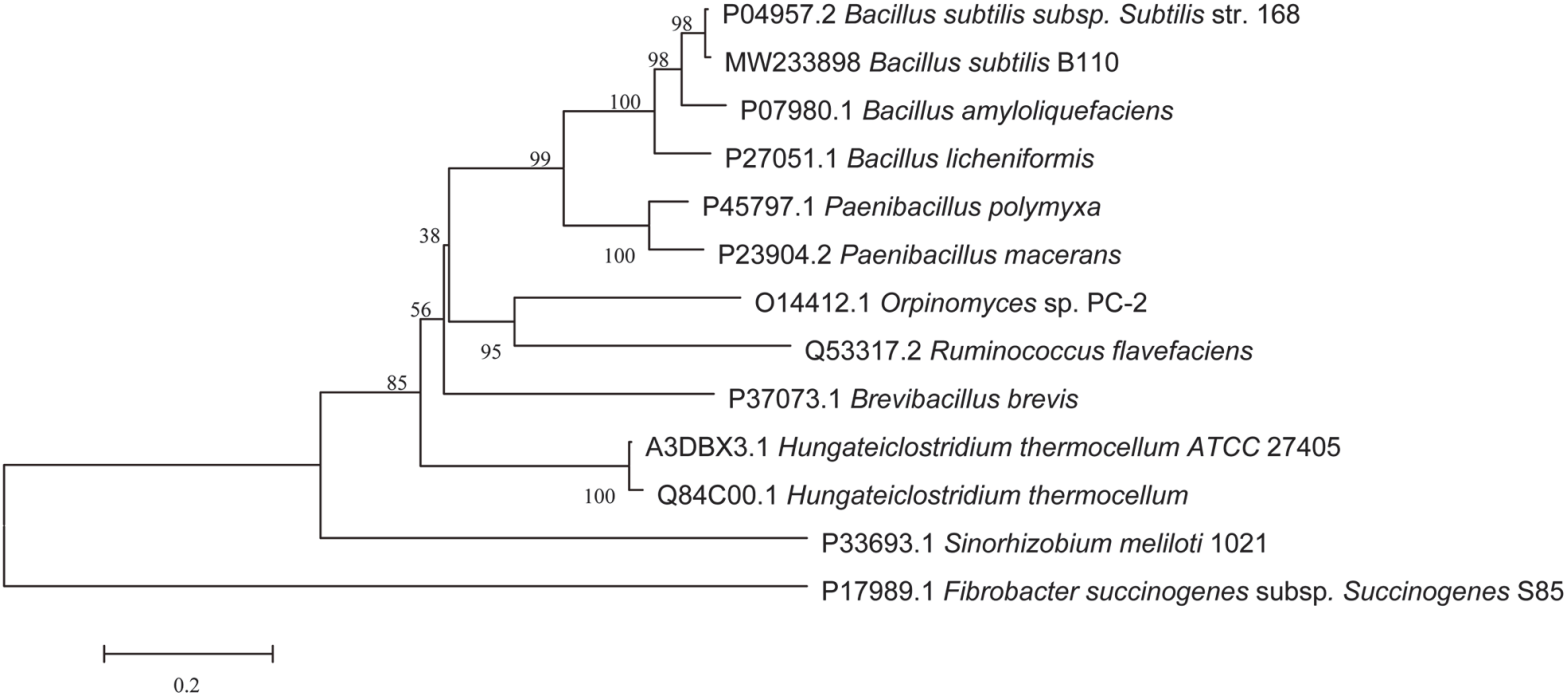
A phylogenetic tree of *CelA203* and related β-1,3-1,4-glucanases from different sources.

The lichenase gene, *CelA203*, was successfully cloned from *B. subtilis* B110. The gene has an ORF of 729 bp encoding a 242-amino acid protein with a predicted molecular mass of 27.3 kDa. The phylogenetic tree (Fig. 1) revealed that *CelA203* clustered on the same branch as *bglS* from *Bacillus subtilis* subsp. 168 (NCBI Accession No. P04957.2). BLAST analysis showed that *CelA203* had a 99.2% identity with 1,3-1,4-beta-D-glucan from *B. subtilis* subsp. 168 [25], followed by *bglA* from *Bacillus amyloliquefaciens* (91.2%, P07980.1) [26], *bgl* from *B. licheniformis* (87.0%, P27051.1) [27], *gluB* from *Paenibacillus polymyxa* (72.5%, P45797.1) [28] and beta-1,3-1,4-glucanase from *Paenibacillus macerans* (70.3%, P23904.2) [29].

### Expression and Purification of CelA203

**Fig. 2:**
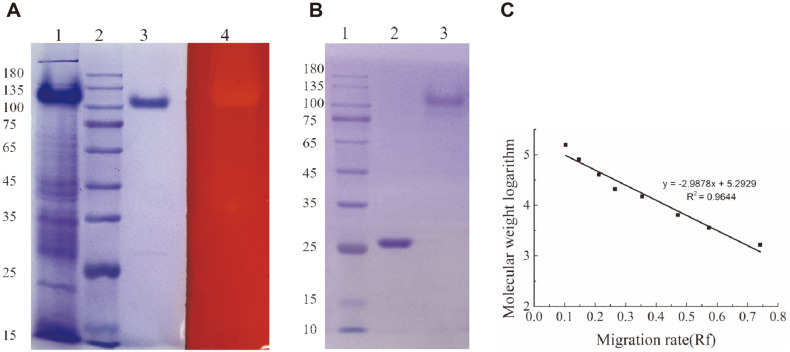
Analysis of expression, purification, zymogram, and molecular weight measurements of CelA203. (**A**) Analysis of expression and purification of CelA203. Lane **1**: supernatant proteins from the recombinant strain; lane **2**: protein marker; lane **3**: purified CelA203; lane **4**: Zymogram analysis of lichanase activity. (**B**) Analysis of boiled and unboiled purified CelA203. Lane **1**: protein marker; lane **2**: boiled purified CelA203; lane **3**: unboiled purified CelA203. (**C**) Migration rate standard curve of proteins of different molecular weight sizes.

The *CelA203* gene was successfully expressed in *E. coli* and SDS-PAGE was employed to confirm the expression of recombinant CelA203. The target protein was mainly in the form of a tetramer (molecular mass 108 kDa)(Fig. 2A). The recombinant CelA203 was successfully purified via Ni-NTA affinity chromatography using different concentrations of imidazole (100, 200, and 300 mM) (data not shown). Purified CelA203 existed in both monomeric and tetrameric forms.

### Zymogram Analysis of Purified CelA203

Results of zymogram analysis of purified CelA203 are displayed in Fig. 2A. Following renaturation and incubation, the CelA203 tetramer in the polyacrylamide gel with barley β-glucan showed an active band when stained with Congo red, whereas the monomer (weak band) displayed a non-active band. The tetramer transformed to a monomer after boiling (Fig. 2B). These findings suggest that the tetrameric form is essential for both the solubility and activity of CelA203. In addition, the protein standard curve (Fig. 2C) was drawn based on the relationship between the molecular weight of the protein and electrophoretic mobility (Rf). Of note, the target polymer protein had a molecular mass of 111.2 kDa, 4.07 times the actual molecular weight of a single protein (27.3 kDa). According to FPLC results, the calculated molecular mass of polymer and monomer were 107.8 and 26.8 kDa, respectively, suggesting the existence of a tetrameric form (Fig. S1).

### Effect of pH and Temperature on the Activity of CelA203

**Fig. 3:**
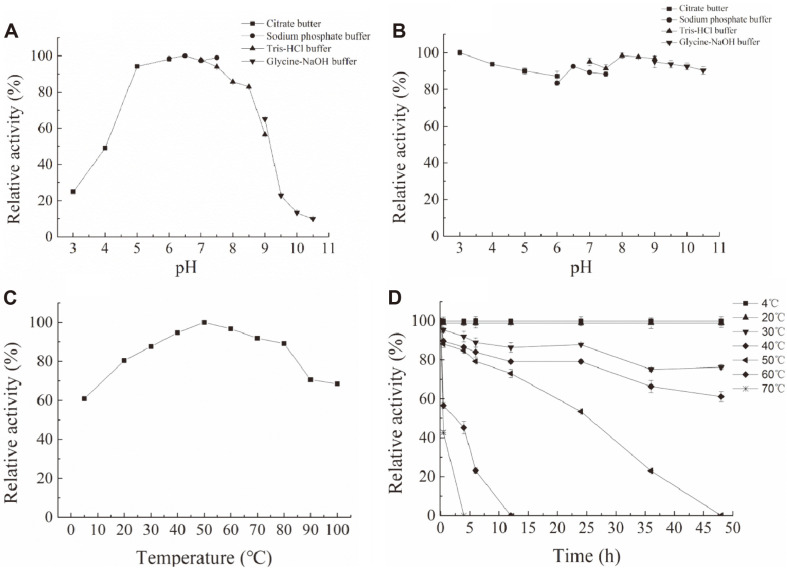
Effects of pH and temperature on the activity and stability of CelA203. (**A**) Optimal pH for CelA203. The optimum pH was determined at 50°C for 5 min, in various pH buffers. (**B**) Analysis of pH stability. Residual activity of CelA203 was assessed under optimum conditions (20 mM PBS buffer, pH 6.5, 50°C, 5 min) after mixing the purified CelA203 with different buffers, at 4°C for 24 h. (**C**) Analysis of the optimum temperature. The optimum activity of CelA203 was examined in 20 mM PBS (pH 6.5) at 4-100°C for 5 min. (**D**) Analysis of thermostability. The activity of CelA203 was assessed by incubating in 20 mM PBS (pH 6.5) at 4-70°C for 48 h.

The recombinant CelA203 exhibited >80% activity at pH 5-8.5, >50% activity at pH 4 and pH 9, and the highest activity was reported at pH 6.5 (Fig. 3A). High activity (>80%) was detected after storage at pH 3-10.5 for 24 h (Fig. 3B). Further analysis showed >60% enzyme activity at 4-100°C and optimum enzyme activity at 50°C (Fig. 3C). Also, the thermal tolerance of CelA203 was measured following incubation at 4-70°C for 48 h (Fig. 3D). CelA203 retained >40% residual activity within 1 h at 70°C, with >50% activity for 4 h at 60°C, and finally >50%activity for 24 h at 50°C. These results provide evidence that CelA203 remains stable in a wide range of pHs and is characterized by favorable thermal stability.

### Effect of Metal Ions and Chemicals on the Activity of CelA203

**Fig. 4:**
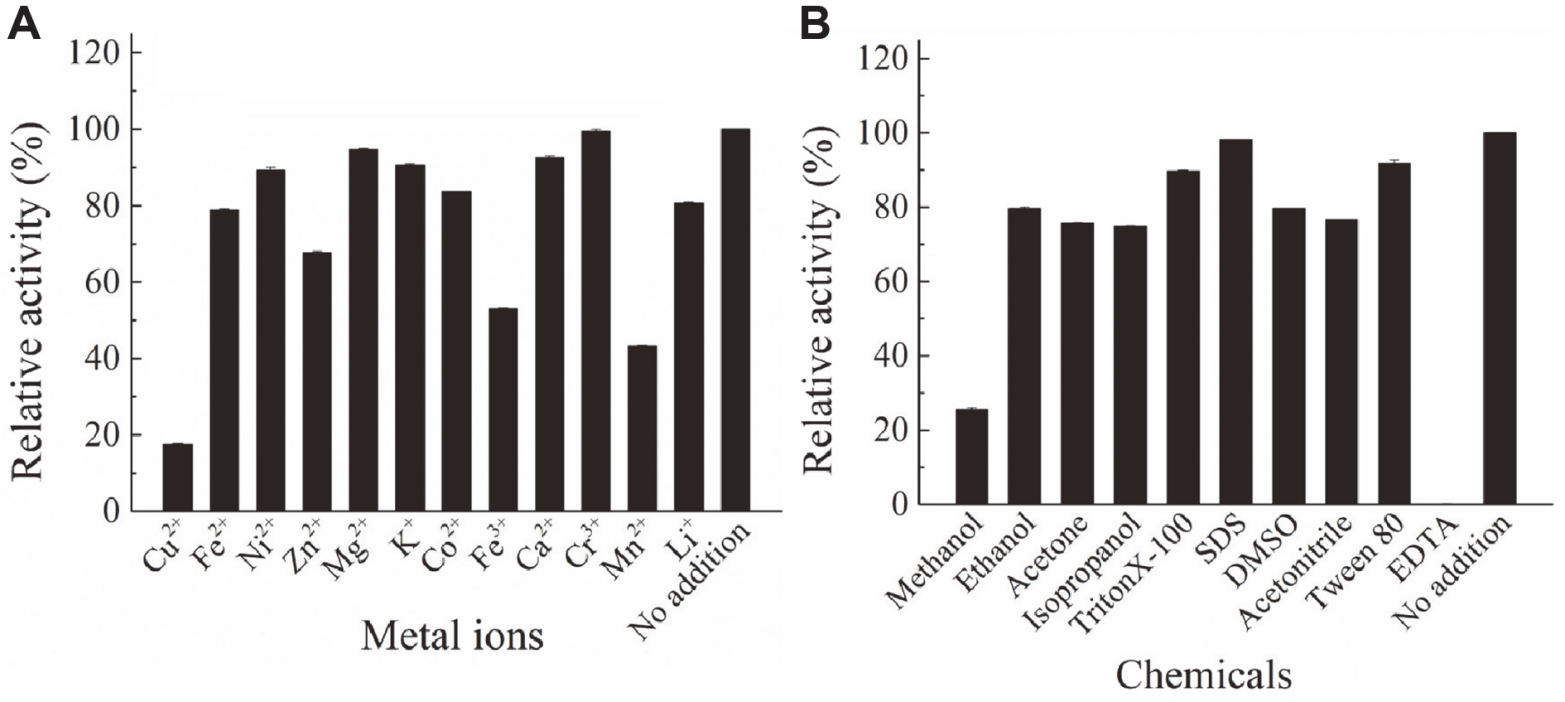
Effect of metal irons (**A**) and compounds (**D**) on the activity of CelA203.

As illustrated in Fig. 4, 1 mM Mn^2+^ and Cu^2+^ strongly inhibited the activity of CelA203 (residual activity <50%), followed by Fe^3+^ (53%), whereas Fe^2+^, Zn^2+^, Li^+^, and Co^2+^ slightly reduced the activity of CelA203 (Fig. 4A). In addition, 10 mM EDTA completely inhibited the activity of CelA203; methanol strongly reduced the activity of CelA203; the other chemicals slightly reduced the activity of CelA203; 10 mM SDS had a negligible effect on the activity of CelA203 (Fig. 4B). We further validated the inhibitory effect of EDTA against CelA203, whereby CelA203 treated with EDTA was dialyzed to remove EDTA. Based on the analysis, 90% of enzyme activity was recovered after the removal of EDTA. In view of these results, the inhibitory effect of EDTA is related to its direct effect on CelA203 and not as a metal ion chelator. The findings suggest that CelA203 is not a metal-dependent hydrolase and EDTA does not chelate the metal ions needed for the activity of the enzyme.

### Substrate Specificity of CelA203

CelA203 demonstrated high specificity towards barley β-glucan and lichenan with a relative activity of 100%(566.38 U/mg) and 30.3% (171.61 U/mg), respectively. On the contrary, CelA203 showed no activity towards laminarin, CMC, xylan, curdlan, avicel, and chitosan (data not shown). The kinetic parameters of CelA203 were also assessed and plotted according to the method described by Lineweaver and Burk. The *K*_m_ and *V*_max_ of CelA203 towards barley β-glucan and lichenan were 3.98 mg/ml, 1017.17 U/mg, and 2.78 mg/ml, 198.24 U/mg, respectively.

### Hydrolytic Properties of CelA203

**Fig. 5:**
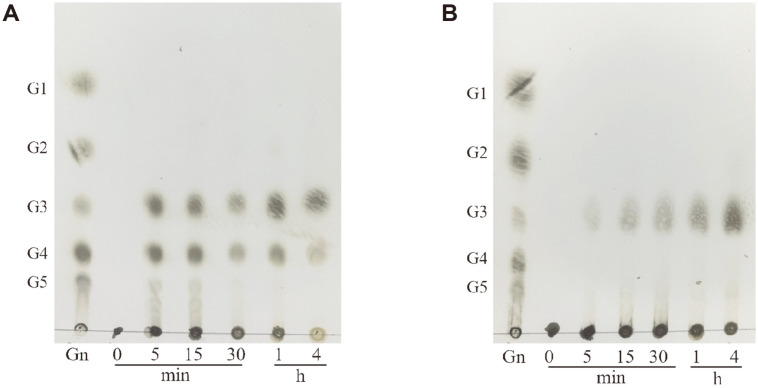
TLC analysis of the CelA203 hydrolysis products from barley β-glucan (**A**) and lichenan (**B**). 1 ml 1% (w/v) of barley β-glucan or lichenan were mixed with 10 U of purified CelA203 in 20 mM PBS buffer (pH 6.5), incubated at 50°C for 4 h after which samples were loaded and analyzed through TLC. (Gn): Standard substances included glucose (G1), cellobiose (G2), cellotriose (G3), and cellotetraose (G4).

The results of TLC analysis of the end products of CelA203 using different substrates are shown in Fig. 5. The main hydrolysis products were trisaccharide and tetrasccharide, and a small amount of higher-molecular-weight oligosaccharides with barley β-glucan as substrate. Notably, the oligosaccharides gradually disappeared with the extension of reaction time. Using lichenan as the substrate, the main hydrolysis products included trisaccharides and a small number of oligosaccharides.

### CelA203-Mediated Hydrolysis of Oat Bran to β-Gluco-Oligosaccharides

**Fig. 6:**
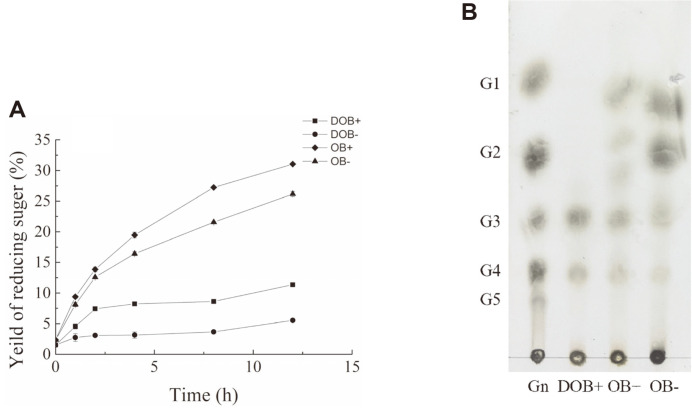
The yield of reducing sugars (**A**) and products of hydrolysis (**B**) following the activity of CelA203 on oat bran or deactivated endogenous oat bran. Purified CelA203 0 or 114 U/g oat bran was added in 5% (w/v) oat bran or deactivated oat bran incubated in 20mM PBS (6.0) buffer at 50°C for 12 h. Reaction mixtures were collected at different intervals and the presence of reducing sugars was verified via the DNS method. Subsequently, the reaction mixtures were boiled for 10 min, after 12 h, and the products were analyzed through TLC. (Gn): Standard substances included glucose (G1), cellobiose (G2), cellotriose (G3), and cellotetraose (G4). DOB+, OB-, and OB+ represent deactivated oat bran with CelA203, oat bran without CelA203, and oat bran with CelA203, respectively.

The amount of reducing sugars from oat bran was shown in Fig. 6A. Following a 12-h incubation, the yield of reducing sugars from oat bran with CelA203 was 31.1%, followed closely by oat bran without CelA203 (26.2%), deactivated oat bran with CelA203 (11.45%), and deactivated oat bran without CelA203 (5.5%). Fig. 6B shows the products of hydrolysis analyzed via TLC. The main products of hydrolysis from oat bran with CelA203 included trisaccharides, tetrasaccharides, and lower-molecular-weight oligosaccharides. The main products of hydrolysis from oat bran without CelA203 included monosaccharides, disaccharides, and a smaller amount of trisaccharides and tetrasaccharides. For deactivated oat bran with CelA203, the main hydrolysis products were trisaccharides and tetrasaccharides.

## Discussion

The applications of β-1,3-1,4-glucanase in feed and brewery industries have, in the recent past, triggered extensive research interest in a bid to improve the conversion of barley-based feed and reduce the viscosity of beer leachate. In this view, several lichenase genes from different sources have been cloned, expressed, and the function of their proteins investigated, such as PbBglu16A from *Paenibacillus barengoltzii* [14], and AaBglu12A from *Aspergillus awamori* [18]. The present study, through BLAST analysis, demonstrated that the lichenase gene, CelA203, has 99.2% identity with the GH family 16 1,3-1,4-beta-D-glucan from *B. subtilis* subsp. 168, with close similariy to *B. amyloliquefaciens*, *B. licheniformis*, and *Paenibacillus polymyxa*. There is a general view that all the lichenases from *Bacillus* species have similar nucleotide and amino acid sequences [7], which explains the high identity between *CelA203* and other *Bacillus* species. The present investigation demonstrated that CelA203 was mainly in a monomeric state in solution, contradicting a previous result on BglS from *B. subtilis* subsp. 168, whose crystal structure exhibited a monomeric form with no description of polymers [9]. Zinc ion binding sites are essential for a protein to form a tetrameric structure [30]. According to our 3D model structure analysis of CelA203 with SWISS-MODEL (template: 1U0A, query cover: 87%, homology: 78.3%), the zinc ion binding amino acids were H173 and D189 (1U0A: H145, D161). Two dimers were interconnected by eight amino acids (Tyr56-Ser53, Ser53-Tyr56, Asn197-Asn200, and Asn200-Asn197).

Compelling evidence has shown the molecular weight range of 25-30 kDa for lichenases from *Bacillus* sp., with monodomain proteins [9, 10, 12], which conforms to the molecular mass of CelA203 (27.2 kDa). On the other hand, non-*Bacillus* β-1,3-1,4-glucanases are larger because of additional domains with different functions. Among them are β-1,3-1,4-glucanases from bacteria, including *C. thermocellum* (38 kDa) [31], *R. flavofaciens* (90 kDa) [32], *Enterobacter* sp. R1 (38 kDa) [33], and *Paenibacillus barengoltzii* (44.0 kDa) [14]. Fungi-derived β-1,3-1,4-glucanases also have high molecular weights, including those from *Talaromyces emersonii* (40.7 kDa) [34] and *Thermomonospora* sp. (64.5 kDa) [35].

Moreover, the activity of recombinant CelA203 was at pH 6.5, consistent with most *Bacillus* sp. lichenases which have been revealed to function at an optimum pH of about 6-7.5, except for *B. brevis* (opt. pH 9) and *Bacillus* sp. N137 (which retains over 80% activity between pH 7 and pH12) [7]. The optimum temperature for CelA203 was 50°C, which agrees with that of lichenases from *B. subtilis* 168 [9] and *B. amyloliquefaciens* ATCC 23350 [36]. CelA203 also remained stable over a wide pH range of 3-10.5, showing a higher residual activity (80%) after incubation for 48 h. Previous evidence had reported similar findings of pH stability for Egl7A from *Talaromyces emersonii* CBS394.64 [13] and β-1,3-1,4 glucanases from *B. licheniformis* UEB CF [37]. In addition, CelA203 retained 40% activity after 1 h incubation at 70°C and maintained 50% of its activity after a 3 h incubation at 60°C. The results demonstrated stronger thermal stability of CelA203 than BglS (catalytic half-life 90 m at 60°C) [9], possibly due to the polymer structure. Of note, the CelA203 activity was maintained at over 80% after the purified CelA203 was stored at 4°C for over one year. We reported stronger thermostability of CelA203 than BGlc8H [38], Egl-257 [39], and lichenase EG1 [37], but lower than that compared to lichenase UEB-S [10] and the glucanase from *Bacillus pumilus* US570 [40]. Furthermore, 1 mM Zn^2+^, Cu^2+^, Fe^3+^, Mn^2+^, and 10 mM EDTA significantly inhibited the activity of CelA203, agreeing with the results reported of β-1,3-1,4-glucanase from *Bacillus altitudinis* YC-9 [12]. Elsewhere, Cu^2+^, Mn^2+^, and EDTA were found to reduce the activity of β-1,3-1,4-glucanase from *B. subtilis* MA139 [41]. The specific activity of CelA203 towards barley β-glucan (566.38 U/mg) and lichenan (171.61 U/mg) was higher than that of GluUS570 (161.87 U/mg and 70.38 U/mg) [8] , and PbBglu16A (425 U/mg and 212 U/mg) [14], but lower than that of TaGlu34 (13,527 U/mg and 9,225 U/mg) [17]. For barley β-glucan, the *K*_m_ value of CelA203(3.98 mg/ml) was similar with that of Egl7A (4 mg/ml) [13] and AaBglu12A (3.51 mg/ml) [18], but the *V*_max_ value (1,017.17 U/mg) was lower than that of both (17,951 U/mg and 12,068 U/mg respectively). CelA203 was shown to possess moderate activity. The *K*_m_ value of CelA203 towards lichenan (2.78 mg/ml) was lower than that of BglS (3.1 mg/ml), which shared the highest amino acid identity with CelA203 [9] and Lic16A (16.88 mg/ml) [15]. Meanwhile, the *V*_max_ value of CelA203 (198.24 U/mg) was lower than that of BglS (2695 U/mg) but higher than that of Lic16A (266.09 U/mg). These data suggested that the tetrameric form of CelA203 shows a higher affinity to lichenan and better thermal stability compared to the monomer.

Generally, bacterial lichenases belong to the GH16 family, whose members are more active towards lichenan and barley β-glucan than members from the other GH families. This is supported by the fact that GH16 lichenases display strict substrate specificity for mixed-linked β-1,3-1,4-glucan. Herein, according to substrate specificity tests, only lichenan and barley β-glucan were hydrolyzed. It is known that the ratio of β-1,4 bonds in barley β-glucans is higher than that in lichenans, contributing to different levels of enzyme activity [42]. Moreover, trisaccharides (3-O-β-cellobiosyl-D-glucose) and tetrasaccharides (3-O-β-cellotriosyl-D-glucose), the main products from the hydrolysis of barley β-glucan, migrated ahead of cellotriose and cellotetraose. On the other hand, tetrasaccharides (3-O-β-cellotriosyl-D-glucose), the main product from the hydrolysis of lichenan, migrated ahead of cellotetraose. These findings identify CelA203 as a lichenase.

Recent years have seen an unprecedented rise in the use of oligosaccharides from different sources for their excellent biological properties, including probiotic, anti-tumor, antioxidant, and immunomodulatory activities [43, 44]. Emerging evidence shows that β-gluco-oligosaccharides have various biological activities, among them, the ability to lower serum cholesterol, antibacterial and antioxidant effects, while also being selective substrates for some probiotics [45]. In a previous investigation, lichenase from *Bacillus circulans* ATCC21367 hydrolyzed barley β-glucan into trisaccharides and tetracose [46]. More evidence demonstrated the direct hydrolysis of oat bran by the β-1,3-1,4-glucanase from *Paenibacillus barengoltzii* [14] and *Thermoascus aurantiacus* [47], yielding the Dp 3-4 and Dp 3-5 oligosaccharides, as the main products, respectively. Consistent with previous findings, the major products from CelA203 hydrolysis of deactivated oat bran, reported in the present work, were trisaccharide and tetrasaccharide.

A lichenase gene (*CelA203*) has been cloned and expressed in *E. coli* successfully. Lichenase exists mainly in the form of the tetramer and demonstrates satisfactory activity over a wide pH range and high thermostability. The findings demonstrate that the lichenase has promising application prospects in the production of oligosaccharides in the animal feed and brewing industries.

## Supplemental Materials

Supplementary data for this paper are available on-line only at http://jmb.or.kr.
