# Supplementary figures and images for: Characterization of a Thermostable Lichenase from Bacillus subtilis B110 and Its Effects on β-Glucan Hydrolysis
Source: J Microbiol Biotechnol. 2021 Dec 15;32(4):484–92. doi: 10.4014/jmb.2111.11017 (PMC9628817; doi:10.4014/jmb.2111.11017)

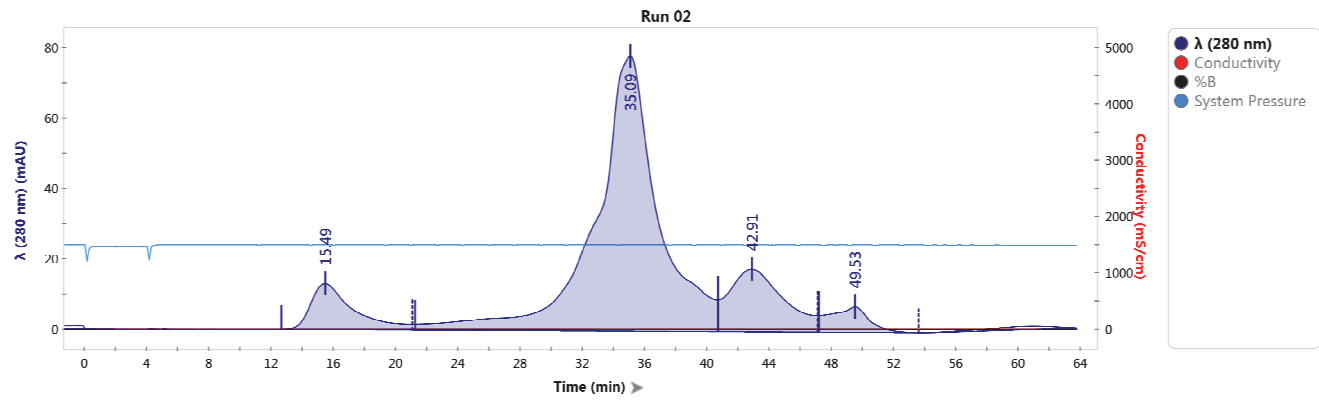

**Fig. S1.** Analysis of fast protein liquid chromatography of purified CelA203.

Supplement: Supplementary file 1 [file jmb-32-4-484-supple.pdf]
